# Overview and Future Advanced Engineering Applications for Morphing Surfaces by Shape Memory Alloy Materials

**DOI:** 10.3390/ma12050708

**Published:** 2019-02-28

**Authors:** Andrea Sellitto, Aniello Riccio

**Affiliations:** Department of Engineering, University of Campania “Luigi Vanvitelli”, 81031 Aversa (CE), Italy; aniello.riccio@unicampania.it

**Keywords:** shape memory alloy, smart structure, preliminary design

## Abstract

The development of structures able to autonomously change their characteristics in response to an external simulation is considered a promising research field. Indeed, these structures, called smart structures, can be adopted to improve the aerodynamic performance of air and land vehicles. In this work, an overview and future applications of Shape Memory Alloys (SMA)-based smart structures are presented. The use of SMA materials seems to be very promising in several engineering sectors. Advanced SMA-based devices, designed to improve the aerodynamic performance of vehicles by modifying the shape of the spoiler and the rear upper panel, are briefly introduced and discussed in this paper. Indeed, a simplified model simulating the SMA mechanical behavior has been considered to demonstrate the feasibility of the introduced smart structures for adaptive aerodynamic applications. Numerical simulations of the investigated structures are provided as a justification of the proposed designs.

## 1. Introduction

The continuously increasing requirements of structures capable of autonomously adapting their shape according to specific varying conditions has led to an increase, in the last few decades, of research studies on morphing technologies. Such technologies are particularly suitable in the aeronautical and automotive fields, where adaptive structures including the development of morphing skin could be employed [1,2,3].

The morphing mechanism can be realized by using smart materials, such as Shape Memory Alloys (SMA), and piezoelectric materials able to modify the shape of the morphing component [4,5,6]. Among the smart materials, SMA are able to recover their initial shape after a deformation has occurred, when subjected to particular thermal conditions [7,8,9]. Moreover, they are characterized by superelastic behavior and a high power-to-weight ratio, which make them particularly suitable for the design of adaptive structures [10]. Several applications related to the adoption of SMA as actuators [11,12,13,14,15,16,17,18,19,20,21], in both the automotive [22,23,24,25,26,27] and aerospace [28,29,30,31,32,33,34,35,36,37,38,39,40,41,42] fields, can be found, even if application on a large scale is still far from being achieved. However, SMA-based actuation systems are being extensively investigated in the framework of several research studies in order to reduce the complexity, with a consequent reduction of weight, of the traditional electric and hydraulic actuation systems [18,43,44]. Indeed, several mechanisms based on SMA actuators are already in use, especially in the sectors of valves and drives, where millions of devices are sold every year [45].

In this work, an overview of smart structures based on SMA actuators is given, together with a preliminary feasibility study on SMA-based actuation devices of vehicle control surfaces. The SMA actuation is finalized to modify the aerodynamic field around the vehicle, by morphing specific key components in order to improve the car performances under varying service conditions. Numerical implementation of the investigated case studies is provided as justification of the feasibility of the proposed design configurations under realistic aerodynamic loads. In Section 2, the state of the art of SMA research and applications is summarized. In Section 3, specific adaptive aerodynamics applications found in the literature are presented. In Section 4, the analyzed case studies are introduced. Finally, in Section 5, the finite element models are described and the results are discussed.

## 2. Overview of the State of the Art of SMA Actuators

Several investigations on morphing concepts, focusing on morphing components, can be found in the literature [46,47,48,49,50,51]. Interesting ideas are provided in [52], where the requirements in the development of morphing surfaces are described, based on previous studies [53,54]. In [55], Smart Soft Composite (SSC) actuators, composed of a woven smart fabric and glass-fiber fabric, were applied to a scaled rear spoiler. The woven smart fabric, consisting of glass fibers with orthogonally placed Ni‒Ti SMA wires within a soft polymer, was combined with additional layers of glass-fiber fabric placed eccentrically with respect to the structure neutral plane. The mechanical properties and the deformations of the resulting SSC can be tailored by modifying the number, orientation, and stacking sequence of both woven and glass-fiber layers. The device was actuated through Joule heating. The different sections of the spoiler can be actuated independently to induce asymmetrical deformations. Experimental tests in wind tunnel were performed on the SSC applied to a 1:8 scale vehicle. The drag force, downforce, side force, and yawning moment induced by bending and bend‒twist deformations were investigated. According to the results, the structure was capable of large deformations while withstanding the external aerodynamic load. Moreover, the structure returned to its initial state once unloaded.

In [56], a comprehensive theoretical and experimental description of an active SMA‒FRP (Fiber-Reinforced Plastic) hybrid structure was presented. A material model, able to accurately predict the SMA actuation behavior, was introduced. The proposed model was validated by comparing the numerical results with experimental data resulting from the experiments. In [57], guidelines for reliable active SMA‒FRP hybrid composite were deduced, based on experimental tests.

Other studies are focused on the modification of the wing geometry by integrating SMA wires in the wing surface [58,59], resulting in an adaptive structure. It is worth highlighting two significant patents. According to the first [60], the wing geometry can be modified by electrically actuating SMA wires embedded within glass fiber skins of a sandwich structure with a honeycomb core. On the other hand, the second patent [61] deals with SMA technologies used to control the curvature of an adaptive wind turbine blade for variable wind conditions.

Additional research can be found, focused on the adoption of the SMA technologies to the development of biologically inspired structures able to mimic the behavior of living beings [62,63,64,65,66]. In [67], the aerodynamic performance of a UAV (Unmanned Aerial Vehicle) morphing winglet, able to mimic the wing-tip deformation of gliding birds, was presented. A smart soft composite, constituted of shape memory alloy wires and glass fibers embedded in a soft polymeric matrix, was used to manufacture morphing winglets that modify their shape without any mechanical device. Preliminary analyses were carried out to determine the flexural stress‒strain relationship by means of three-point bending tests, taking into account various SMA wire diameters and glass-fiber layers. Moreover, the effects, in terms of winglet end-edge deflection, of adopting different SMA wire diameters, different volume fraction of the embedded SMA, and different glass-fiber layers in the winglet were assessed. In order to evaluate the aerodynamic coefficients, experimental tests were conducted in an open-blowing wind tunnel, considering different angles of attack. The morphed geometry was found to improve the L/D ratio to 5.8%.

In [68], the development of smart components for advanced aircraft systems is presented. SMA hybrid composite panels were considered thanks to their superior performance in terms of thermal buckling and post-buckling behavior, fatigue, dynamic, and structural acoustic response. The aim is to manufacture SMA-based composite panels for the reduction of the sonic fatigue in aeronautic structures. Moreover, in [69,70,71,72,73] the superior performance in terms of energy absorption and impact response of Shape Memory Alloy Hybrid Composites (SMAHC) is assessed. Other applications of SMA can be related to SMA wires embedded in complex wing structures [74,75,76], while in [77], SMA wires interact with a supportive system of pins and springs to actuate an articulated control surface.

Further studies focused on the analytical [78,79,80,81,82,83,84,85,86], numerical [59,65,66,79,81,87,88,89,90,91,92,93], and experimental [54,59,65,66,78,79,80,90,91,92,93,94,95,96,97,98,99,100,101] investigation of SMA-based smart structures. Indeed, SMA were used as axial [91], flexural [54,65,66,93,98,99,100], twisting [94,95], or non-planar [79,96] actuators.

An experimental/numerical investigation on a device capable of multiple actuation modes was introduced in [102]. The device was composed of four SMA wires embedded in a PDMS (polydimethylsiloxane) soft matrix. One or two SMA wires can be activated at once. Since the SMA wires are positioned at a negative or a positive eccentricity with respect to the middle plane of the device, the actuation resulted in an out-of-plane displacement. Hence, activating the different SMAs was able to induce bending mode, twisting mode, or a combination of bending and twisting modes. Experimental tests were performed to measure the deflection and the twisting angle of the device during the actuation of the different SMA wires. According to the experimental tests, deformations up to 160°, in both the pure bending and twisting modes, were observed. Moreover, the same device was able to deform up to 80° for both bending and twisting in the combined mode. Finally, finite element simulations were presented to predict the device behavior in terms of mode, direction, and deformation magnitude. Tanaka-based models [103] were used to numerically simulate the SMA thermomechanical behavior. The numerical results were found to be in agreement with the experimental ones.

Important studies on SMA modeling can be found [104,105]. In particular, in [104] one-dimensional thermodynamics and statistical thermodynamics models for a crystalline body, characterized by an austenitic phase and martensitic twins, were developed. In [105], an overview of SMA actuators in smart structures was presented, focusing on their modeling and simulation.

In [106], the mechanical properties of unsymmetrical smart composite laminates were experimentally determined. The investigated laminates were composed of two layers: a unidirectional carbon fiber epoxy laminate and a SMA wire epoxy laminate. The final structure was able to bend under an applied thermal load. Four configurations were analyzed and characterized by different spatial densities of the SMA wires, to assess their influence on the mechanical behavior of the specimens. Conventional tensile machines were found to be unsuitable for evaluating the mechanical properties of the laminate due to the asymmetry of the specimens. Therefore, a specially developed tensile testing machine was used for asymmetrical materials. According to the experimental results, the mechanical properties of the laminate are slightly enhanced by increasing the SMA wires density.

In [107], the relationship between stress and deformation in a composite structure with embedded SMA wires was investigated. The influence of SMA wires in a composite plate and the reliability of the actuation of hybrid composites by means of shape memory alloys were assessed. SMA Ti‒Ni wires embedded in epoxy resin were considered. Experimental tests were conducted on the specimens by applying a tensile external load. Moreover, the strength of the SMA‒matrix interface was experimentally determined by means of pull-out tests. The tests were performed at different temperatures: lower than the austenite start temperature and higher than the austenite finish temperature, to completely characterize the SMA mechanical behavior. Numerical analyses were performed to simulate the SMA behavior by means of the superelastic shape memory material model available in LS-Dyna.

The actuating ability and reliability of small hysteresis SMA hybrid composites were studied in [108], where basic guidelines for the design of SMA hybrid composites were provided based on experimental studies. In particular, the investigated hybrid laminate consisted of pre-strained TiNiCu wires coupled with glass and Kevlar fibers epoxy prepreg. It was found that the alloy is characterized by very small hysteresis during the thermal cycle. Moreover, the actuating potential of the considered SMA wires is not negatively affected by the curing process, up to 413 K. The study also focused on the effect of the SMA pre-strain. In particular, high pre-strain was found to result in high internal stress, which both weakens the SMA wires/matrix interface and reduces the actuation ability of the laminate. The debonding can be delayed by adding fibers with negative thermal expansion coefficient, like Kevlar ones. Finally, the working temperature of the SMA laminate was required to be lower than the glass transition temperature of the matrix and the debonding temperature of the interface.

Other works on the behavior of 3D adaptive structures composed of reinforced plastic fibers based on shape memory alloys can be found in the literature [7,109,110,111]. In particular, experimental investigations of hybrid yarn-based actuators with SMA cores were carried out in [112]. Several parameters of the structure have been considered for the experimental tests, in order to determine the spatial deformation behavior of the 3D actuator.

However, designing with SMA can be very challenging, due to their limitations. Indeed, one of the most cumbersome issue encountered when designing with SMA is related to their actuation and de-actuation speeds. In [113], a large electrical current was used to improve the actuation speed of SMA-based actuators by increasing the heating rate. Moreover, the size (diameter) of the SMA wire plays a fundamental role in the actuation speed [114]. Indeed, lower-diameter wires are characterized by higher external surface/volume ratios, increasing the heating and cooling speeds. In [115], the actuation frequencies of different SMA materials were investigated. The frequency was increased by using different active cooling systems, such as thermal gel, flowing air, heat sinking and forced air, and fluid quenching.

Furthermore, limitations related to the fatigue life, which affects the durability and the reliability of SMA devices, must be addressed. In [116], the effects of the stress level, of the thermal cycling temperature interval, and of the heat-treatment state on the fatigue-life performances of TiNi wires were investigated. According to the study, the fatigue life is strongly influenced by the temperature interval adopted during the thermal cycling. The fatigue life of SMA wires is also strongly influenced by the stress and strain reached in their actuated state, as suggested in [117], where SmartFlex NiTi wires subjected to cyclic tensile loads were experimentally investigated. To reduce the thermal and mechanical overstresses induced in actuated SMAs, bi-stable configurations were investigated [118,119,120,121,122] to develop mechanisms able to shift between a stable de-actuated configuration and a stable actuated configuration. Hence, the activation of the SMA is needed to actuate and de-actuate the devices, resulting in energy-free actuation states where an electric current is not needed to keep the device in its actuated configuration, reducing the power consumption and the thermal and mechanical overstresses as well. Moreover, in [123] considerations related to the low energy efficiency of SMA actuators ware addressed. Different load cases for SMA actuators were compared, resulting in an efficiency that ranges between 0.013% and 1.3%.

## 3. Adaptive Aerodynamic Applications

Adaptive aerodynamics is one of the most promising fields of applications for shape memory alloy components, thanks to their morphing capabilities. To date, several solutions have been investigated to modify the aerodynamic field in aeronautical applications. In [124], a stretchable UAV wing able to increase its planform area by 80% is presented. Shape Memory Polymers (SMP) were used to modify the wing chord, to tailor the wing to specific scenarios. The adoption of actively cooled SMA for the deployment of flexible control surfaces was investigated in [48], while in [96] SMA wires were used to modify the camber of a morphing wing. Experimental and numerical investigations on a morphing airfoil were carried out in [58], where SMA springs were used to actuate discrete points of the structure to achieve the desired deformation. In [125], SMP hinges, adopted to modify the sweep angle of a wing, were numerically and experimentally investigated, while in [126] the thickness of a flexible skin morphing wing is controlled by means of SMA actuators. A feasibility study of wing flap actuation based on shape memory alloys is presented in [127]. A wing composed of different telescopic segment deployed by means of SMA actuators is presented in [29] to improve the aerodynamic performances while reducing the wing volume stowage. In [128,129], SMAs were used to design and manufacture smart vortex generators, and their performance was investigated by means of experimental wind tunnel tests. In [38], an application to deploy and stow a flap edge fence is presented. 

**Figure 1:**
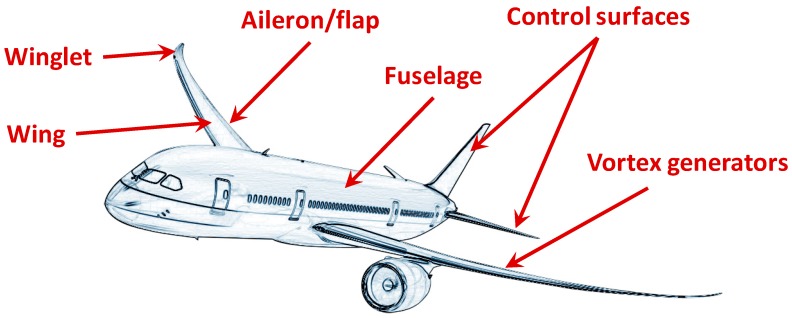
Locations of adaptive aerodynamic applications in the aeronautical field.

Figure 1 summarizes the present and future applications of SMA concepts for adaptive airplanes aerodynamics.

**Figure 2:**
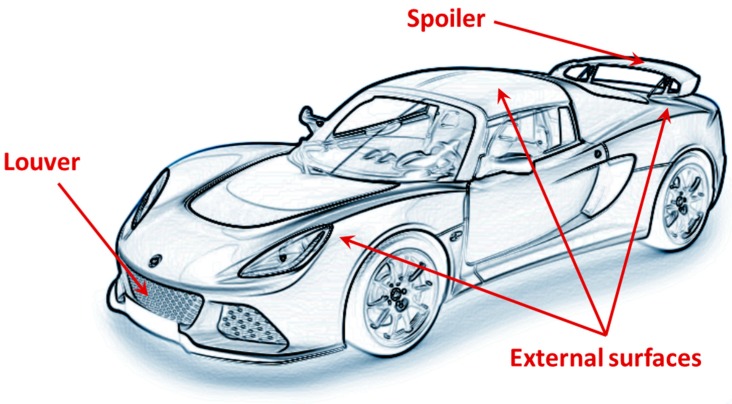
Locations of adaptive aerodynamic applications in the automotive field.

The solutions developed for adaptive aerodynamics can be easily transferred from the aeronautical to the automotive field. In the automotive field, adaptive aerodynamic applications include, but are not limited to, actuation of external surfaces, spoilers, and/or grill/louvers, as shown in Figure 2.

In this work, two preliminary case studies, finalized to the adaptive aerodynamic, are briefly introduced. The presented case studies aim to demonstrate feasibility and inspire future applications to develop SMA-based devices in the adaptive aerodynamics field. Hence, in this preliminary design stage, the SMA characteristics have not been taken into account; instead we focus on the load exerted during the actuation. Indeed, in an advanced design stage, more detailed analyses must be performed, supported by experimental data. In the proposed case studies, SMA actuators have been adopted to modify the aerodynamic field of vehicle on demand. The presented case studies are focused on the spoiler and on the rear upper panel components.

## 4. Description of Case Studies

### 4.1. Case Study #1—Trailing Edge Actuation

**Figure 3:**
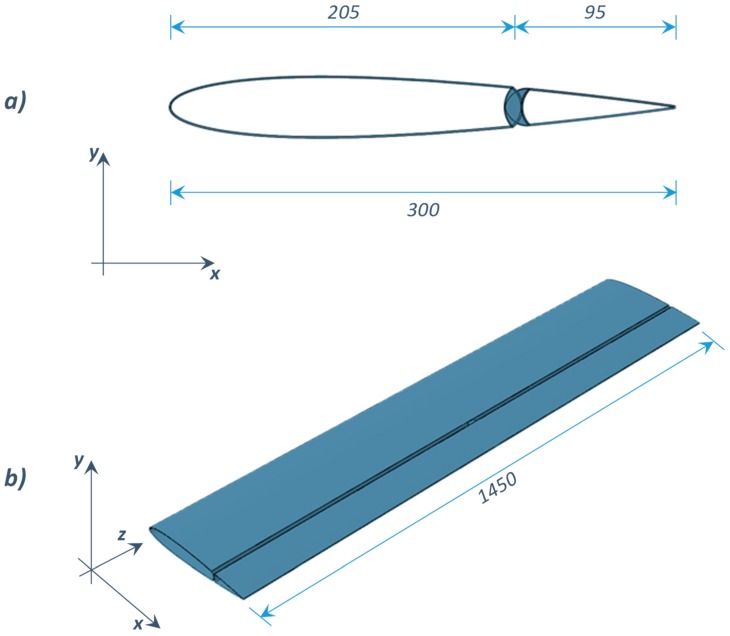
Spoiler and actuated elevator: (**a**) cross section; (**b**) isometric view (unit: mm).

The aim of Case study #1 is to demonstrate the feasibility of SMA-based actuation of a spoiler trailing edge under service aerodynamic loads. A NACA 0012 airfoil has been considered as the spoiler section, as shown in Figure 3.

The trailing edge actuation will result in a variation in drag and downforce. From preliminary aerodynamic studies, a requirement of at least 10 mm trailing edge displacement should be achieved to guarantee a significant variation of the aerodynamic field.

### 4.2. Case Study #2—Rear Upper Panel Actuation

**Figure 4:**
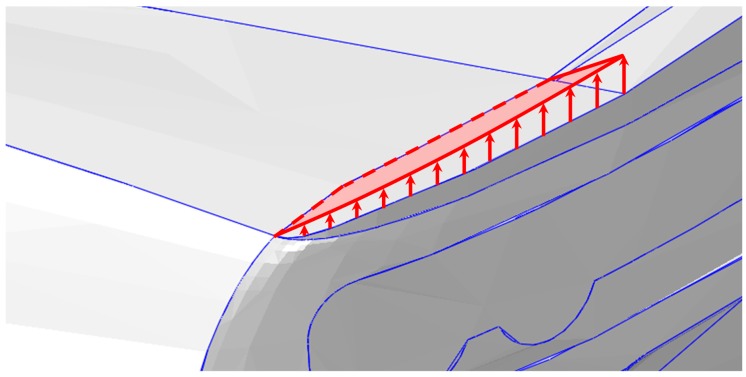
Rear panel actuation.

The aim of the second case study (see Figure 4) is to demonstrate the feasibility of the actuation of rear upper panels of a vehicle, inducing a variation of the aerodynamic field, with consequent variation of drag and downforce to improve the vehicle performance under service aerodynamic loads. In order to perform realistic numerical computations, the geometry configuration of an existing sports car has been taken into account. From preliminary aerodynamic studies, a 10 mm displacement of the rear upper panel has been considered sufficient to significantly modify the aerodynamic field.

## 5. Numerical Simulation

### 5.1. SMA Modeling

**Figure 5:**
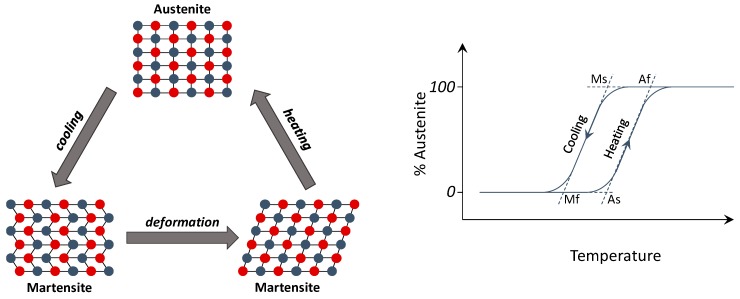
(**Left**) NiTiNOL transformation; (**right**) austenite variation as a function of the temperature.

The Shape Memory Alloys are metallic alloys able to recover an initial prescribed shape when subjected to temperature variation. In particular, an increase of temperature beyond a prescribed threshold generates a phase transition from a martensitic crystal structure to a stable austenitic crystalline structure, as shown in Figure 5. Since the phase transition induces a rearrangement of the microstructural arrays, the SMA mechanical properties including the elastic modulus and the yield strength are significantly modified [130,131,132,133].

Among the other SMAs, NiTiNOL, an alloy based on nickel and titanium (Nickel Titanium Naval Ordnance Laboratory), is one of the most used and investigated.

In this work, a NiTiNOL wire is modeled in ABAQUS by means of linear 3D beam B31 elements. In this phase, a 100 mm long wire, characterized by a 1 mm diameter circular profile representative of a bundle of SMA wires, is considered. A NiTiNOL Ni_52_Ti_48_ alloy [134] has been taken into account. Table 1 reports the mechanical properties of the SMA material system.

A simplified SMA material model has been adopted. This approach, although not accounting for all the characteristics of the SMA such as the hysteresis, can still be used in a preliminary study to evaluate the load resulting from the SMA actuation. Indeed, the main advantage of this approach is the simplicity of implementation in commercial codes, since it only requires defining temperature-dependent material properties, such as elastic modulus *E* and Coefficient of Thermal Expansion (CTE) *α*. However, in a more advanced design stage, detailed SMA constitutive models [135,136,137,138,139] must be used to assess the behavior of the SMA actuators.

According to [140,141], the variations of both the elastic modulus *E* and the CTE *α* are taken into account as the temperature increases. In this work, the experimental data found in [134,141] have been used. In particular, the elastic moduli and the coefficients of thermal expansion needed to describe the behavior of the alloy were derived from a database of experimental tests, exploiting the calibration proposed in [142]. In particular, the elastic moduli *E* was experimentally measured from isothermal tensile tests: for each desired temperature, the SMA wires were subjected to a tensile load at a constant temperature. Moreover, the CTEs *α* were experimentally evaluated by applying an increasing thermal load on a pre-strained SMA wire (4% in the current study). Hence, the CTE at different temperatures can be expressed as a function of the measured strains and temperatures.

In order to numerically replicate the SMA characterization procedure, as a preliminary step to the case studies analyses, a NiTiNOL wire has been clamped at its extremities. An initial temperature equal to 25 °C has been assumed, while the elastic modulus and the thermal expansion coefficients adopted have been changed with temperature according to Table 2.

**Figure 6:**
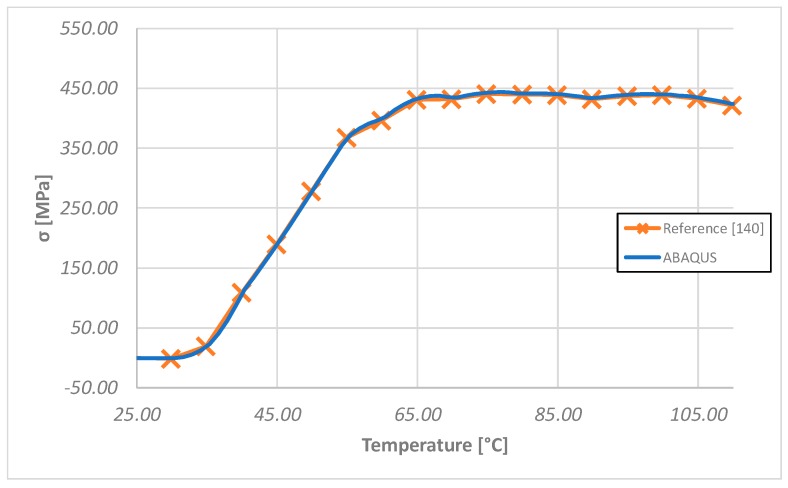
Numerical test results [140].

This analysis is aimed at the validation of the proposed simplified material model with respect to the literature data. Indeed, it does not describe the behavior of the SMA wire used as an actuator, due to the different boundary conditions. The results of the ABAQUS numerical test, in terms of stress as a function of the temperature, have been found to be in agreement with the data reported in [140], as shown in Figure 6, where the numerical test results are compared to the reference ones. Thus, the validated material model has been used in the following test cases, tailoring the number of SMA wires according to the specific application.

### 5.2. Case Study #1

**Figure 7:**
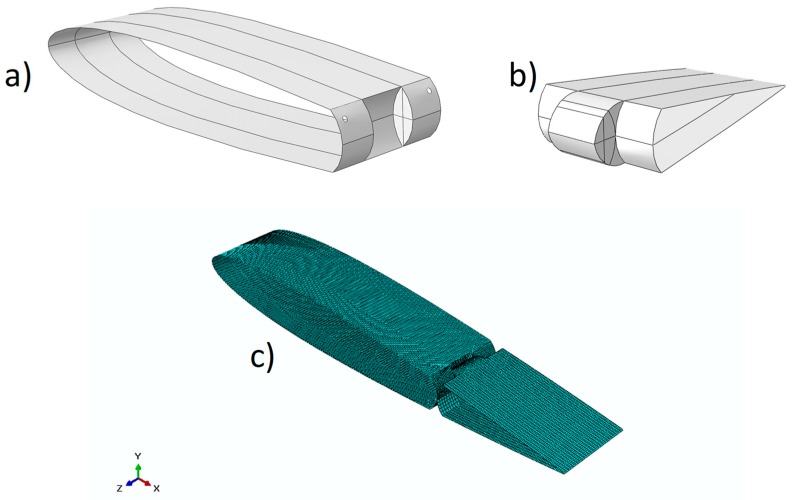
Case study #1 (details): (**a**,**b**) geometries; (**c**) FEM.

In Case study #1, NiTiNOL wires have been integrated into an aluminum spoiler structure. According to the proposed finite element discretization, two solid models (Figure 7a,b) have been considered. The first model represents the fixed part of the spoiler, while the second represents the moving tip (see Figure 7). The two solid models have been discretized by means of four-noded shell elements with a reduced integration scheme (S4R). In Figure 7, details of both solid models with corresponding numerical discretization are shown, while the mechanical properties of the adopted aluminum alloy have been reported in Table 3.

**Figure 8:**
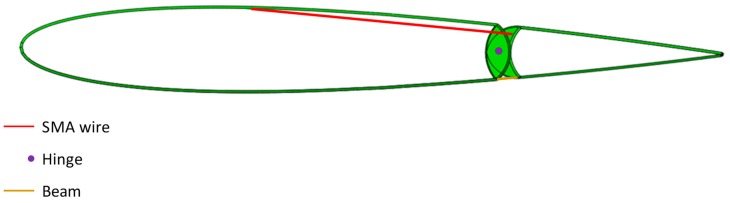
Trailing edge actuation, details of the SMA, hinge, and beam locations.

A hinge placed between the fixed and the moving part allows the rotation of the tip. As already mentioned, two 120-mm-long SMA wires have been introduced to connect the fixed part to the moving tip, through two holes drilled on the fixed part (see Figure 7a). The position of the SMA wires has been chosen to maximize the moment with respect to the hinge. The section of the wires and the properties of the material are defined in agreement with the preliminary study on SMA materials. In order to guarantee the return of the device to its initial position at SMA deactivation, two elastic beams have been placed between the fixed and moving tip opposed to the SMA wires. Details of the SMAs and beams locations are reported in Figure 8.

**Figure 9:**
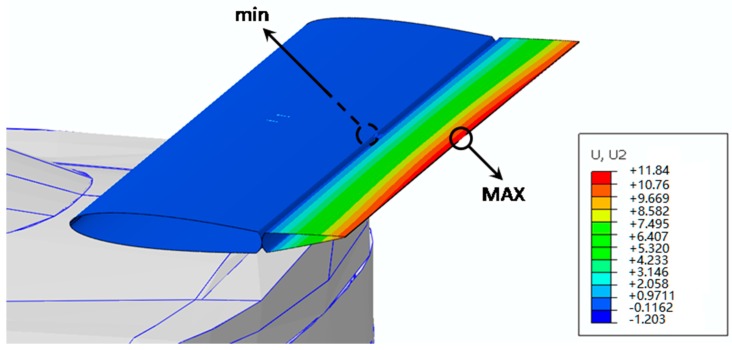
Case study #1: actuated structure (unit: mm).

An initial 25 °C temperature has been defined for the whole model. The SMA wires have been actuated by increasing their temperature to 150 °C. Two different analyses have been considered. The first analysis has been finalized to the testing of the SMA wire mechanical behavior and the latter has been finalized to the investigation of the influence of the external aerodynamic load and the elastic load of the beams on the SMA mechanical behavior and on the SMA actuation. Indeed, an equivalent aerodynamic load of 2.95 kg, evaluated by means of preliminary aerodynamic simulations, has been applied on the moving tip. Since the weight is one order of magnitude lower than the aerodynamic load, it has been neglected. In Table 4, the results of both configurations are reported, while Figure 9 shows Case study #1 in actuation mode with the application of both the aerodynamic load and the elastic load from the beams. The maximum values of stress and strain observed during the actuation in the SMA wires are 250 MPa and 10^4^ με, respectively. According to the numerical results, the investigated device can satisfy the displacement requirement (10 mm). 

### 5.3. Case Study #2

**Figure 10:**
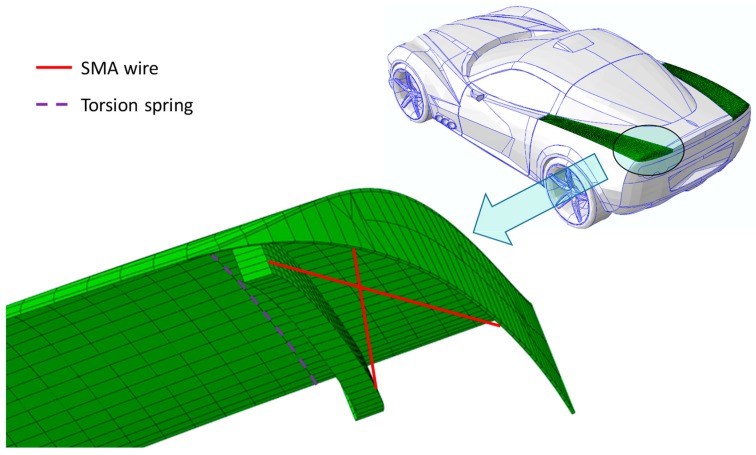
Rear upper panel actuation, details of the numerical model, and locations of SMA and torsion spring.

Case study #2 is focused on the actuation of the rear upper panel of a bonnet, by means of properly located 270-mm-long NiTiNOL wires, aimed to increase the drag and the downforce. The numerical model has been realized by means of eight-noded 3D solid elements with a reduced integration scheme (C3D8R). A hinge has been placed between the fixed part of the bonnet and the rear upper panel interfaces to allow its rotation. The rear upper panel can recover its initial position by means of a torsion spring placed in the hinge. A 1.2 kg equivalent aerodynamic load, evaluated by means of preliminary aerodynamic simulations, has been considered on the moving surface. As in the previous case study, the weight has been neglected since it is one order of magnitude below the aerodynamic load. An initial temperature equal to 25 °C has been defined on the whole model, and the SMA wires have been actuated by increasing their temperature up to 150 °C. In Figure 10, the numerical model, including the location of the SMA wires and of the torsion spring, is reported.

**Figure 11:**
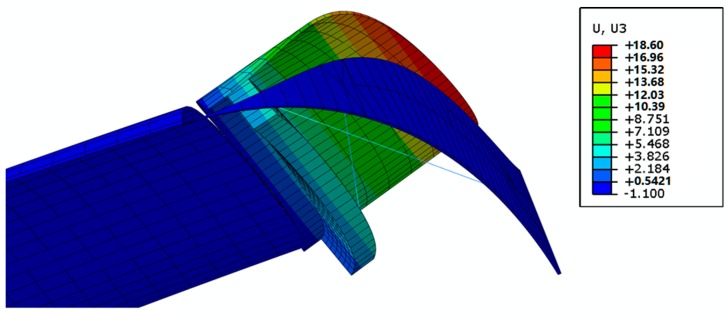
Case study #2: actuated structure. (unit: mm).

Finally, in Figure 11, the structure in actuation mode is reported. Again, for this configuration, the requirement on displacements (10 mm) needed to obtain a significant aerodynamic field variation has been satisfied. The maximum values of stress and strain observed during the actuation in the SMA wires are 350 MPa and 400 με, respectively. However, it is worth noting that modifications to the number and the length of the SMA wire can help to tailor actuation displacements.

## 6. Conclusions and Discussion of Future Trends

**Figure 12:**
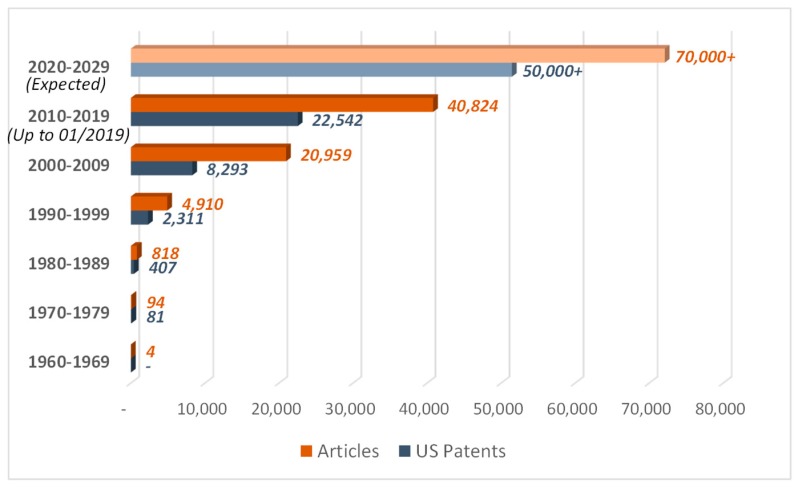
Articles published and U.S. patents issued. Sources: Scopus and Uspto. Keywords: “Shape Memory Alloy” OR “Nitinol”. Retrieved: 22/01/2019.

An overview of SMA-based smart structures has been presented in this work. Extensive studies, analytical, numerical, and experimental, have been found in the literature dealing with shape memory alloys, which can be considered suitable for adaptive aerodynamic applications, thanks to their morphing capabilities. Indeed, the research efforts on SMA-based actuators have been focused on the development of devices able to induce axial, bending, or twisting deformations. Moreover, the adoption of shape memory alloys results in simpler and lighter devices compared to the conventional actuators, significantly reducing the weight and the cost of the components. Thus, the interest in shape memory alloy applications is increasing even more, as demonstrated by the number of articles published and patents issued, as reported in Figure 12.

**Figure 13:**
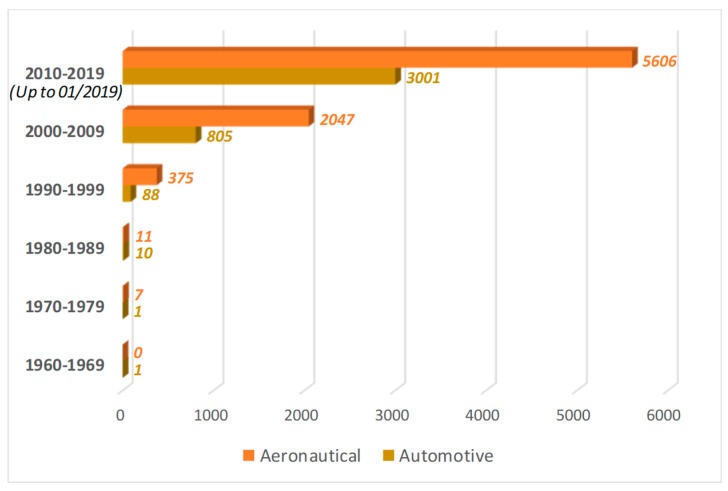
Articles published in the aeronautical and automotive fields. Sources: Scopus. Aeronautical keywords: (“Shape Memory Alloy” OR “Nitinol”) AND (“Aerospace” OR “Aeronautical” OR “Flight” OR “Aircraft”); Automotive keywords: (“Shape Memory Alloy” OR “Nitinol”) AND (“Automotive” OR “Car” OR “Vehicle”). Retrieved: 22/01/2019.

As a matter of fact, the majority of the applications presented in the literature are confined to the aerospace field, where performance requirements are demanding, as shown in detail in Figure 13, where the number of articles clearly belonging to the aeronautical or automotive fields are compared.

Hence, based on this literature research, a feasibility study of the development of SMA-based smart actuators for automotive applications, mostly derived from the aerospace experience, has been presented in this paper. In order to focus on the feasibility rather than on the executive design of SMA concepts, in this work a simplified model, descriptive of the NiTiNOL characteristics, has been employed. Moreover, with the same objective in mind, preliminary, if realistic, aerodynamics loads have been considered for the proposed SMA concepts. Two case studies have been presented: the trailing edge actuation of a spoiler (very close to the aerospace background) and the rear upper panel deformation of a vehicle. The provided numerical analyses have demonstrated the feasibility of the presented SMA-based smart devices. As a general remark, the key design parameters to be considered in SMA applications, such as the maximum attained force and displacement and the operating range temperature, can be controlled by varying the material, size, and shape of the adopted SMA wires. Hence, the presented solutions can be improved by tailoring the SMA geometry and material characteristics.

## Figures and Tables

**Table 1:** Ni_52_Ti_48_ mechanical properties [134].

A_s_ (°C)	A_f_ (°C)	M_s_ (°C)	M_f_ (°C)	E^A^ (GPa)	E^M^ (GPa)
41.23	69.60	19.67	3.91	68	21

**Table 2:** SMA temperature-dependent mechanical and thermal properties.

Temperature (°C)	E (MPa)	α (°C^−1^)
24	21,259	6.61 × 10^−6^
30	19,905	6.61 × 10^−6^
35	21,303	−1.8777 × 10^−4^
40	21,483	−5.1203 × 10^−4^
55	38,346	−3.8596 × 10^−4^
60	43,625	−3.0564 × 10^−4^
70	55,325	−1.9657 × 10^−4^
80	57,519	−1.5404 × 10^−4^
90	55,440	−1.3077 × 10^−4^
110	58,790	−9.0464 × 10^−5^
150	57,750	−5.9532 × 10^−5^

**Table 3:** Al2024-T6 mechanical properties.

E (MPa)	ν (-)
72,300	0.33

**Table 4:** Results of Case study #1.

Boundary Conditions	Max Displacement (mm)
Without load and beam	16.03
With load and beam	11.84
